# Monitoring the immune response of macrophages in tuberculous granuloma through the expression of CD68, iNOS and HLA-DR in naturally infected beef cattle

**DOI:** 10.1186/s12917-023-03763-5

**Published:** 2023-10-21

**Authors:** Mohamed G. Hamed, Jaime Gómez-Laguna, Fernanda Larenas-Muñoz, Abdelzaher Z. Mahmoud, Fatma Abo Zakaib Ali, Sary Kh. Abd-Elghaffar

**Affiliations:** 1 Department of Pathology and Clinical Pathology, Faculty of Veterinary Medicine, Sohag University, Sohag, 82524 Egypt; 2 Department of Anatomy and Comparative Pathology and Toxicology, Pathology and Immunology Group (UCO-PIG), UIC Zoonosis y Enfermedades Emergentes ENZOEM, University of Córdoba, International Excellence Agrifood Campus ‘CeiA3’, Córdoba, 14014 Spain; 3 Department of Pathology and Clinical Pathology, Faculty of Veterinary Medicine, Assuit University, Assiut, Egypt; 4 Department of Pathology and Clinical Pathology, School of Veterinary Medicine, Badr University in Assiut, Assiut, Egypt

**Keywords:** Tuberculosis, Granuloma, Macrophage, CD68, iNOs, HLA-DR

## Abstract

Bovine tuberculosis still represents a universal threat that creates a wider range of public and animal health impacts. One of the most important steps in the pathogenesis of this disease and granuloma formation is the phagocytosis of tuberculous bacilli by macrophages. Mycobacteria replicate in macrophages, which are crucial to the pathophysiology of mycobacterial infections; however, scarce information is available about the dynamics of the granuloma-stage immunological response. Therefore, immunohistochemistry was used in this work to evaluate the expression of CD68, iNOS, and HLA-DR in different stages of TB granulomas from naturally infected cattle with tuberculosis. Two thousand, one hundred and fifty slaughtered beef cattle were examined during the period from September 2020 to March 2022. Sixty of them showed gross tuberculous pulmonary lesions and samples were collected from all of them for histopathological examination, Ziehl-Neelsen (ZN) staining, and bacteriological culturing. Selected samples that yielded a positive result for ZN and mycobacterial culturing were subjected to an immunohistochemical study of CD68, iNOS, and HLA-DR expression by macrophages according to granuloma stages. Immunohistochemical analysis revealed that the immunolabeling of CD68^+^, iNOS^+^, and HLA-DR^+^ macrophages significantly reduced as the stage of granuloma increased from stage I to stage IV (*P <* 0.003, *P <* 0.002, and *P <* 0.002, respectively). The distribution of immunolabeled macrophages was similar for the three markers, with immunolabeled macrophages distributed throughout early-stage granulomas (I, II), and surrounding the necrotic core in late-stage granulomas (III, IV). Our results suggest a polarization to the pro-inflammatory environment and increased expression of CD68^+^, iNOS^+,^ and HLA-DR^+^ macrophages in the early stages of granulomas (I, II), which may play a protective role in the immune response of naturally infected beef cattle with tuberculosis.

## Introduction

*Mycobacterium tuberculosis complex* (MTC) is the primary cause of bovine tuberculosis (bTB), which affects a variety of domestic, wild animal species, and humans [1]. Cattle are considered the main reservoir of the disease, which also poses a zoonotic risk, infecting humans. Animals with TB are significantly less productive, which results in significant economic losses [2, 3]. According to the World Health Organization (WHO), the disease causes 3 million human deaths and 8 million new cases caused by *Mycobacterium tuberculosis* each year [4]. The disease geographic distribution disproportionately varies within countries and across the globe, and poverty is the strongest predictor of incidence [5]. In Africa, where insufficient diagnosis and treatment are exceedingly widespread, the disease has a particularly high incidence rate, accounting for 25% of all global TB cases [6]. Sudan is one of the developing countries where tuberculosis is a major public health issue, with an estimated 29,000 cases in 2019 [7]. The most characteristic lesion of bovine tuberculosis is the formation of granulomas in target organs, more significantly in the lungs, lymph nodes, intestines, kidneys, and others [8, 9].

Experimental infections have made it possible to qualitatively classify granulomas into four stages (I-IV) in cattle based on size, cellular composition, and the presence or absence of necrosis, fibrosis, and mineralization in granulomas during the course of bovine tuberculosis infection [10, 11] which has also been characterized in natural infections [12, 13]. Despite the granuloma’s significance as a physical barrier in the immune response against *M. bovis*, the dynamics of the granuloma-stage immunological response are scarcely understood [14]. Mycobacterial immunity is predominantly a cell-mediated immune (CMI) response that involves the activation of macrophages, dendritic cells, and T helper type 1 (Th1) cells that are controlled by cytokines [15, 16]. Mycobacteria are phagocytosed by and replicate in macrophages, which are crucial to the pathophysiology of mycobacterial infections. In this sense, the quantity and location of macrophages have been reported to vary as the granuloma developed. Macrophages make up a significant proportion of the cell population within stage I and stage II granulomas, however, they often form a thin rim around the necrotic core in stage III and stage IV granulomas and are less prevalent in the outermost layers of the connective tissue capsule of the granuloma [11].

Macrophages are one of the first immune cells to interact with inhaled bacilli, and they can suppress bacterial growth through phagocytosis, phagolysosome fusion and acidification, lysosomal proteolytic enzymes, and the production of reactive oxygen and nitrogen species with antimicrobial properties [17]. Two distinct phenotypes can be found within the granuloma. Firstly, mycobacteria are eliminated more quickly by activated macrophages within a pro-inflammatory environment. Secondly, an anti-inflammatory milieu leads to an alternative activation of macrophages, intended to preserve tissue integrity and support tissue repair [18]. Interestingly, whereas a proinflammatory environment is found close to the necrotic center, anti-inflammatory signals are situated more peripherally close to the capsular region in human tuberculous granulomas [19]. Nonetheless, there is scarce information about how this spatial distribution in bovine tuberculosis.

CD68 has been widely employed as a pan-macrophage marker or an M1 marker [20–22]. CD68 is a member of the family of molecules known as lysosome-associated membrane proteins, consisting of a glycosylated type I membrane protein and being connected to the phagocytic activity of the cell [23]. Inducible nitric oxide synthase (iNOS)a marker for classically activated macrophages (M1), is one of the most intriguing markers regarding macrophage polarization [24, 25]. For the generation of Nitric oxide (NO), which can eradicate mycobacterial species. When iNOS is stimulated, inactivates mycobacteria by making highly reactive nitrogen intermediates (peroxynitrite) [26]. iNOS uses arginine as a substrate. iNOS is known to be crucial for the management of *M. tuberculosis* infection in mice [27, 28] and *M. bovis* in bovine [27, 28]. L-arginine is converted by the enzyme iNOS into L-citrulline and NO in the presence of oxygen, which is linked to the elimination of the infection and the destruction of intracellular mycobacteria [24, 29, 30]. Another marker of interest to characterize macrophage subpopulations is class II MHC antigens (MHC-II). MHC-II is crucial for controlling immunological responses and antigen presentation, particularly for T cell-mediated immune response [21]. The expression of MHC-II can be assessed by an antibody against HLA-DR, which has specificity for the α–a chain of human MHC-II and cross-reacts with bovine tissues [31, 32].

Understanding the immunopathogenesis of bovine tuberculosis requires extensive research and new approaches. Therefore, comprehension of host-pathogen interactions at the granuloma level is crucial. In the current study, immunohistochemistry (IHC) was used to investigate the expression of CD68, iNOS, and HLA-DR within different stages of TB granulomas in naturally tuberculosis-infected cattle. The role and spatial distribution of immunolabeled macrophages are also discussed.

## Materials and methods

### Animals, postmortem examination, and specimen collection

Regular visits to the Middle East and Wadyna slaughterhouses were carried out from September 2020 to March 2022. A routine postmortem examination of 2,150 male beef cattle (Zebu) aged from 2 to 3 years was carried out with particular attention to tuberculous affections. The current study was conducted on beef cattle imported from Sudan. The prevalence of zoonotic human bTB is seven cases/100,000 population/year in Sudan [33]. Sixty out of 2,150 animals presented gross tuberculosis-like lesions (TBL) and tissue samples from tracheobronchial and mediastinal lymph nodes were collected from each animal.

### Histopathological examinations

Tracheobronchial and mediastinal tissue samples from the 60 animals with TBLs were subjected to histopathological examination and granuloma staging. Tissue samples were fixed in 10% neutral buffered formalin for 24 to 72 h, embedded in paraffin, sectioned into 4 μm sections, and stained with hematoxylin-eosin (H&E) and Ziehl-Neelsen (ZN) acid-fast stain. A sample was considered positive for ZN when one or more acid-fast bacilli (AFB) were noticed in at least one high-power field magnification (HPF, 100x) of the sample and accordingly the lesions were classified as paucibacillary (1 to 10 AFB per HPF), or pluribacillary (≥ 11 AFB per HPF) [34]. Granulomas were classified into four different stages (stages I to IV) according to the previously described criteria [10, 13].

### Bacteriological culture and real-time PCR targeting IS6110 of MTC

Samples were collected from tuberculosis like lesions (N = 60) for bacterial culture. On a petri plate, very fine cuts were performed on sample with a scalpel. The Petroff method, which was modified by adding 4% NaOH, was used to decontaminate the samples. The suspension was agitated for 15 min, centrifuged for 15 min at 3,000 rpm, the supernatants were discarded, and water was added for rinsing. Re-centrifugation at 3,000 rpm carried out for 15 min. Löwenstein Jensen (LJ) medium was inoculated from the sediment. The bottles were incubated at 37 °C until mycobacteria proliferation was noticed, and then stored at room temperature. Mycobacteria were detected using the nitrate reduction test after the culture was certified positive. Deoxyribonucleic acid (DNA) extraction from tissue samples (N = 10) and real-time PCR targeting IS*6110* was performed. Specific primers (IS6110-forward: 5′ GGTAGCAGACCTCACCTATGTGT- 3′; IS6110-reverse: 5′ AGGCGTCGGTGACAAAGG-3′) targeting a conserved region of IS6110 transposon were used. The diagnostic performance of the qPCR was conducted using the QuantiFast^R^ Pathogen PCR + IC Kit according to the previously described criteria [35].

### Immunohistochemical examinations

For the immunohistochemical study, the tuberculous granuloma was considered as the experimental unit. Five representative samples from five animals were selected, all of them presenting a positive result to ZN staining and mycobacterial culture. A minimum of 10 granulomas per stage was required for the subsequent immunohistochemical study. The avidin-biotin-peroxidase complex (ABC Vector Elite; Vector Laboratories) was used to analyze the expression of CD68, iNOS, and HLA-DR antigens in the different stages of the granulomas. Briefly, 4 μm tissue sections were deparaffinized and rehydrated through graded alcohols, followed by blocking endogenous peroxidase activity using 3% hydrogen peroxide in methanol for 30 min in darkness. Table 1 shows the antigen recovery method and primary and secondary antibodies. After antigen retrieval, sections were washed with phosphate buffer saline (PBS) (pH 7.4) and incubated with blocking solution for 30 min at room temperature in a humidity chamber. Primary antibody was applied and incubated overnight at 4 °C. To establish the absence of non-specific binding, a negative control was included replacing the primary antibody with the corresponding blocking solution, depending on the antibody. Following a PBS wash, the appropriate biotinylated secondary antibody was applied for 30 min, followed by the Avidin-Biotin-Peroxidase Complex (Vector Laboratories), which was then incubated for 1 h at room temperature in darkness. Labeling was visualized by using the NovaRED™ substrate kit (Vector Laboratories). Finally, slides were mounted, dehydrated, and counterstained with Harris hematoxylin.

**Table 1:** Summary of immunohistochemical methodology

Primary antibody (Clone)	Type	Dilution	Supplier	Antigen retrieval	Blocking solution	Secondary antibody (dilution)
CD68 (EBM11)	Monoclonal anti-human	1:100	Dako	Protease from *Bacillus licheniformis* 0.64% in PBS for 8 min	BSA 1%	Anti-Mouse (1:200)
iNOS	Polyclonal Rabbit	1:500	NeoMarkers, Fremont CA	Citrate pH 6.0 oven	NGS 10%	Anti-Rabbit (1:200)
HLA-DR antigen Alpha-chain, clone TAL.1B5	Monoclonal anti-human	1:20	Dako	Citrate buffer pH 3.2 in microwave	NGS 10%	Anti-Mouse (1:200)

iNOS: inducible nitric oxide synthase; HLA-DR: Human leucocytic antigen-DR; BSA: Bovine serum albumin; NGS: Normal Goat serum; Anti-Mouse: Polyclonal Goat-Anti-Mouse (Dako, Santa Clara, USA); Anti-Rabbit: Goat Anti-Rabbit IgG (Vector Laboratories, USA)

### Immunohistochemistry evaluation

The immunolabeled sections were examined by light microscopy. In each slide, immunolabeled cells were identified within the different stages of tuberculous granuloma. The percentage of area covered by immunolabelled cells was determined by digital image analysis (Image J software) after setting the.

thresholds. Necrotic or mineralized areas in stage III and stage IV granulomas.

were not included in the analysis, as described previously [11]. The results are expressed as the percentage of the positively immunolabeled area within the total area of the granuloma.

### Statistical analysis

The results of the immunohistochemical analyses were expressed as the mean and standard deviation (SD) and the results were compared between the different stages of granuloma. A *P* value < 0.05 was considered statistically significant. The analyses were conducted using GraphPad Prism 5.0 software (GraphPad Prism software 5.0, Inc., San Diego, CA, USA). One-way analysis of variance (ANOVA) and Tukey’s multiple comparison post hoc test was used to compare expression between different stages of granuloma.

## Results

### Pathological examination

**Fig. 1:**
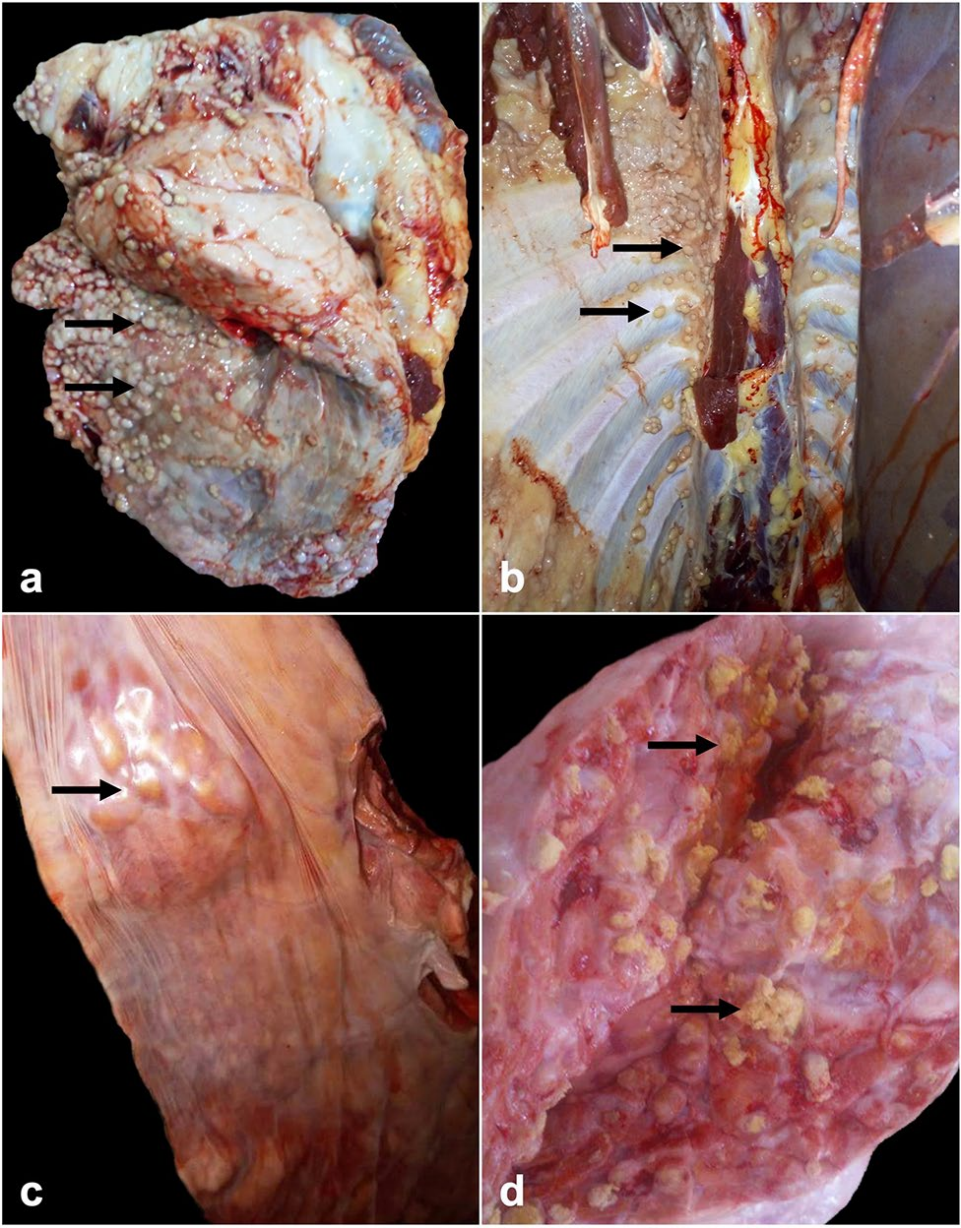
Lung of cattle: (**a&b**): large number of small gray to white yellowish tubercles (pearl tuberculosis) above visceral and the parietal pleura (**arrows**). (**c&d**): multiple nodular lesions (**c, arrow**); cut sections revealed cheesy necrotic material (**d, arrows**) with calcification

**Fig. 2:**
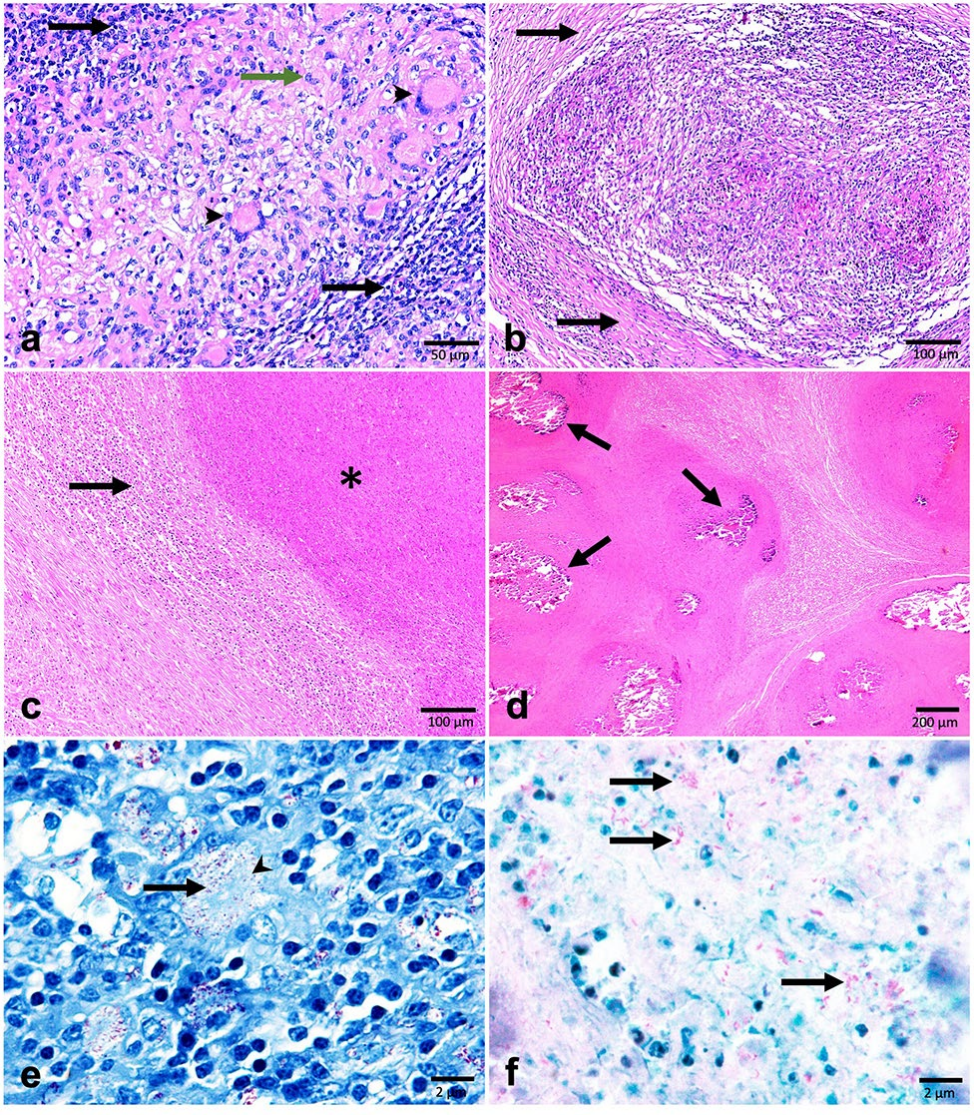
Histological sections of bronchial lymph nodes showing the four stages of granulomas from naturally infected beef cattle with bovine tuberculosis. **(a): Stage I granuloma**, epithelioid cell (green arrow), and multinucleated giant cells (**arrowheads**) surrounded by lymphocytes (arrows). (Hx **&** E. Bar = 50 μm). **(b): Stage II granuloma** (solid), increased numbers of epithelioid macrophages can be seen, encircled by thin layer of C.T. **(arrows**), and central caseous necrosis is not formed (Hx & E, Bar = 100 μm). **(c): Stage III granuloma** showing fully formed C.T. (**arrows**) and central caseated area (**asterisk**) with little mineralization (Hx**&** E, Bar = 100 μm). (**d): Stage IV granuloma** with an extensive central caseated area, mature C.T capsule, and extensive multiple mineralizations (**arrows**) (Hx& E, Bar = 200 μm). **(e)**: Intracellular acid-fast bacilli present inside the multinucleated giant cell (ZN stain) **(arrowhead). (f**): a large number of acid-fast bacilli **(arrows)** were detected in the center necrotic area of the granuloma

Sixty out of the 2,150 examined cases presented tuberculous lesions (2.79%). Based on gross observations, these lesions were grouped into 9 (15%) cases diagnosed as generalized tuberculosis and 51 (85%) cases diagnosed as localized tuberculosis. Affected lungs, pleura, and peritoneum of generalized cases were characterized by miliary tuberculosis with a large number of small gray to white-yellowish miliary nodules resembling pearls (Fig. 1a and b). However, in the localized cases, variably sized multinodular lesions were detected, with cheesy necrotic material or creamy inspissated pus when sectioned (Fig. 1c and d). According to the histopathological examination the granulomas were classified into 4 stages (I, II, II, I, and IV) (Fig. 2a and d). A total of 515 granulomas were evaluated from tracheobronchial and mediastinal lymph nodes. The distribution of the 515 cattle granulomas according to the granuloma stage was: 110/515 (21.35%) stage I; 122/515 (23.68%) stage II; 98/515 (19.0%) stage III, and 185/515 (35.92%) stage IV. Forty-two out of 60 animals (70%) yielded a positive result with the ZN technique, with 35 cases showing a paucibacillary form, whereas 7 cases showed a pluribacillary form (Fig. 2f). AFB was mainly demonstrated within macrophages, multinucleated giant cells (MNGCs) and the necrotic center of granulomas (Fig. 2e and f).

### Bacteriological culture and real-time PCR targeting IS6110 of MTC

Of the total 60 cultures, 35 cultures were positive. *Mycobacterium bovis* was confirmed by a negative result of nitrate reduction test which is characteristic of *Mycobacterium bovis.* The 10 samples which subjected to real-time PCR targeting of IS6110 of mycobacterium tuberculosis complex yielded a positive result. These 10 samples also yielded a positive result to bacteriological Culturing.

### Distribution of CD68 immunolabeled cells

**Fig. 3:**
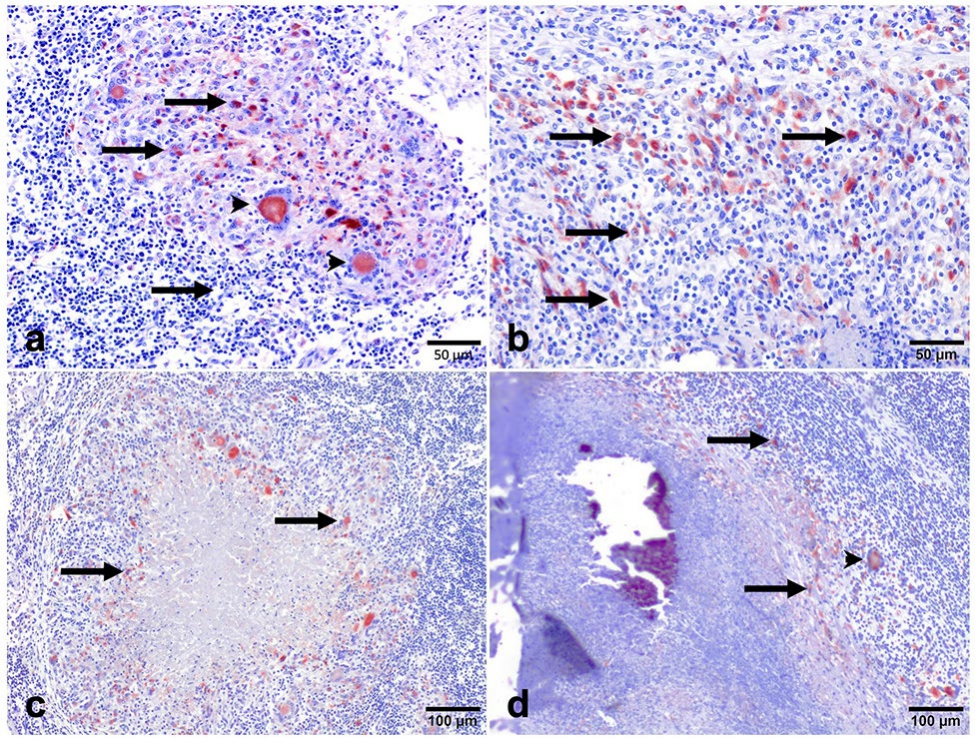
Expression of macrophages (CD68^+^). CD68^+^ staining in (**a**): stage I and (**b**): stage II granulomas in the lymph node of beef cattle naturally infected with *Mycobacterium*. Heavy positive staining can be observed within the cytoplasm of macrophages (arrows) and multi-nucleated giant cells (arrowheads). (IHC, 50 μm). (**c**): Scattered CD68^+^ macrophage cells can be detected within the rim of inflammatory cells surrounding the necrotic center of the stage III granuloma (arrows). (**d**): Expression of CD68^+^ macrophages in a stage IV granuloma (arrows). (IHC, 100 μm). The expression of CD68^+^ macrophages can be seen within the cytoplasm of a few epithelioid, macrophages, and multi-nucleated giant cells (arrow head) surrounding the necrotic center. (IHC, 100 μm)

**Fig. 4:**
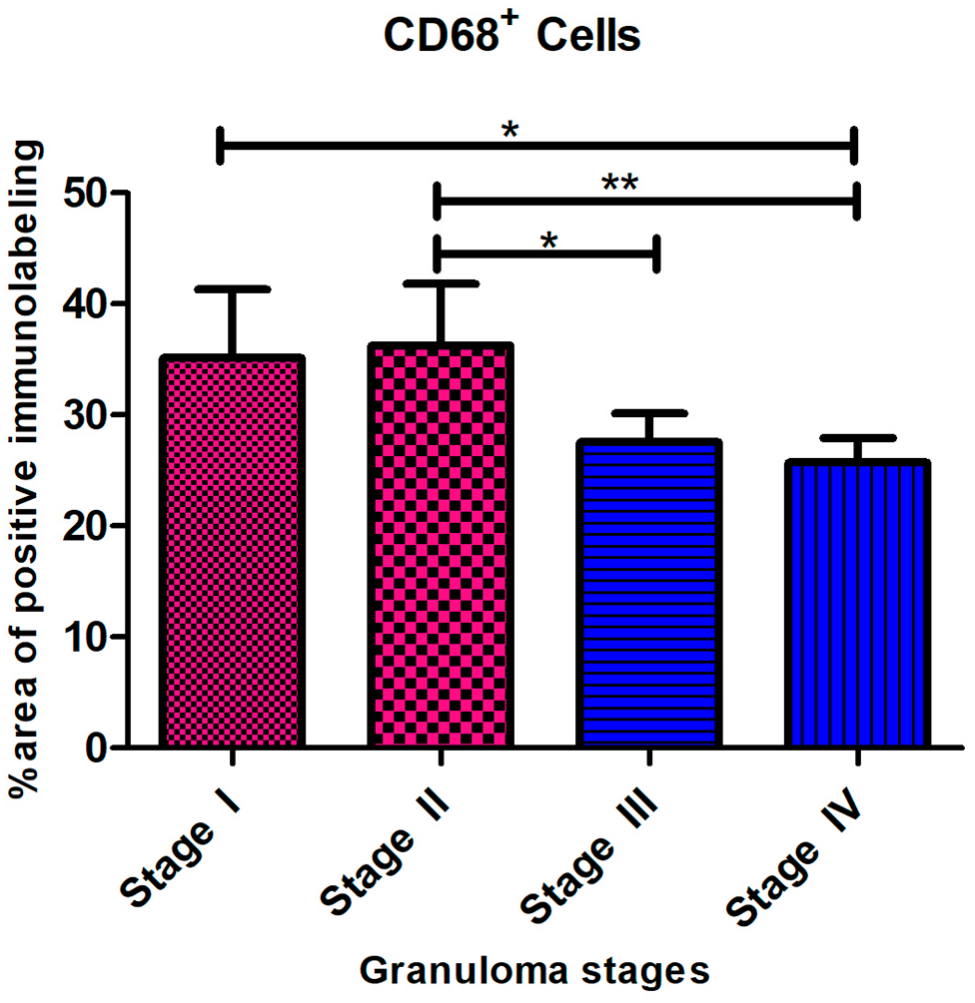
CD68^+^ Cells. The CD68^+^ immunolabeling fraction was higher in the early-stage granulomas compared with the late stages, with a significant difference between stage II and stages III and stage IV (** = *P < 0.003)*

Immunohistochemistry for CD68 showed diffuse cytoplasmic expression in macrophages, epithelioid macrophages, and Langhans MNGCs in all stages of granuloma. The intensity of staining was strong in stage I and stage II granulomas. The location of these cells differed between stages, thus, while CD68^+^ macrophages were dispersed throughout stage I and stage II granulomas (Fig. 3a and b), in stages III and IV there was an obvious rim of positively stained macrophages surrounding the necrotic center (Fig. 3c and d). The number of CD68^+^ cells decreased as granuloma development occurred (** = *P < 0.003)* (Fig. 4).

### Distribution of iNOS immunolabelled cells

**Fig. 5:**
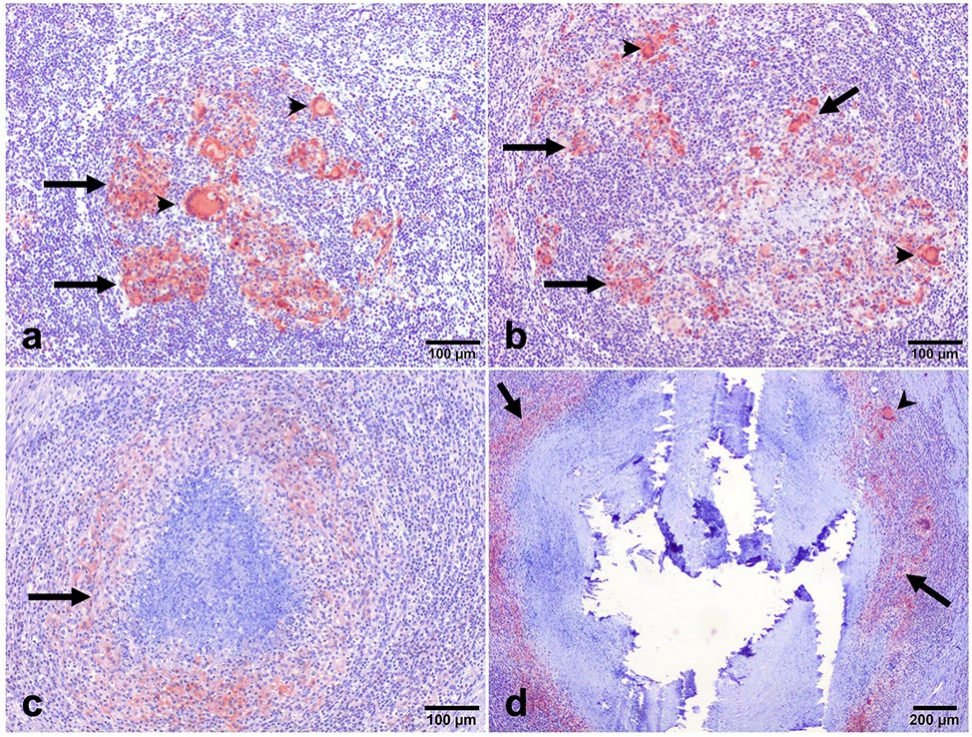
Expression of iNOS^+^ immunolabeling in (**a**): stage I and (**b**): stage II granulomas in the lymph node of beef cattle naturally infected with *Mycobacterium*. Strong positive expression was observed within the cytoplasm of macrophages (arrows) and multi-nucleated giant cells (arrowheads). (IHC, 100 μm). (**c**): iNOS^+^ macrophages forming a peripheral rim surrounding the necrotic center of the stage III granuloma (arrows). (**d**) Expression of iNOS^+^ macrophages in a stage IV granuloma (arrows). (IHC, 100 μm). The expression of iNOS^+^ macrophages (arrowhead) can be seen surrounding the necrotic center in the outer layer of the granuloma. (IHC, 200 μm)

**Fig. 6:**
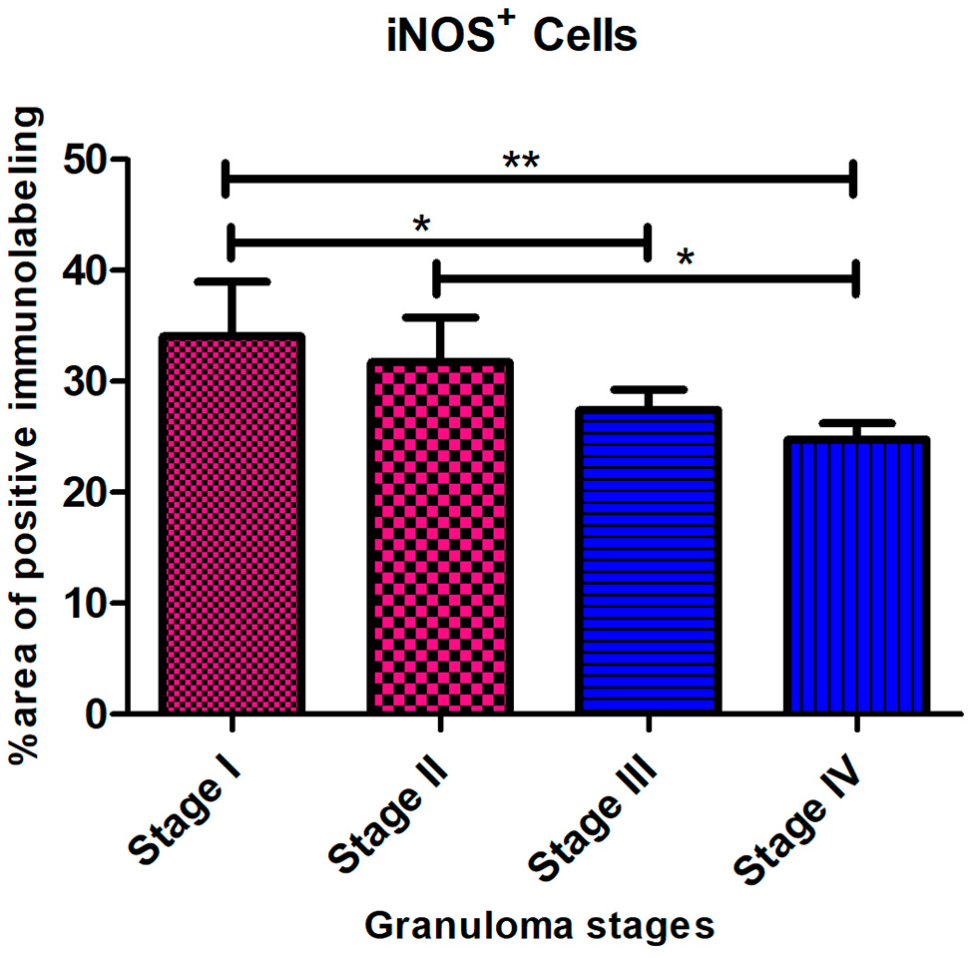
iNOS^+^ Cells. The iNOS^+^ immunolabeling fraction was higher in the early-stage granulomas compared with the late stages, with a significant difference between stage I and stage IV (** =*P < 0.002)*

Anti-iNOS antibody was used to identify M1 macrophagesiNOS-positive cells consisting of macrophages, epithelioid cells, and MNGCs, with an intense cytoplasmic expression observed especially in the latter. The intensity of immunolabelling was higher in stage I and stage II granulomas, mainly within epithelioid macrophages and MNGCs in the center of the granuloma (Fig. 5a and b), whereas stage III and stage IV granulomas showed a rim of iNOS^+^ macrophages surrounding the necrotic center (Fig. 5c and d). Regarding the kinetics of expression of iNOS, the mean frequency of positive cells was higher in the early stages of granulomas compared to the late stages of granulomas (** =*P* < 0.01; stage I vs. stage IV granulomas) (Fig. 6).

### Distribution of HLA-DR immunolabeled cells

**Fig. 7:**
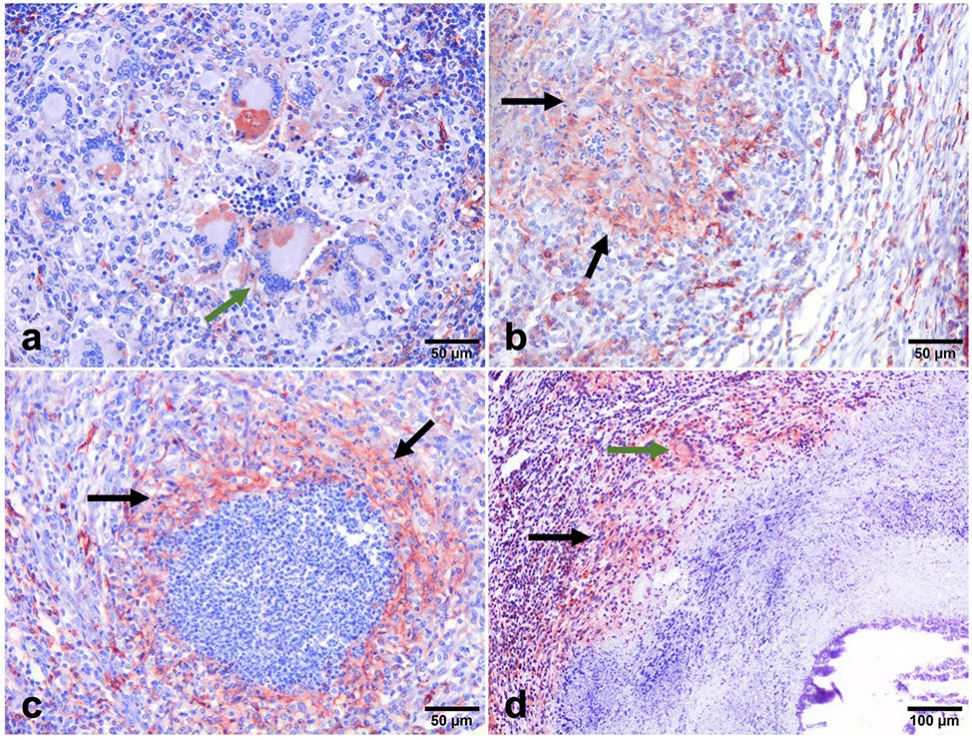
Expression of macrophages (HLA-DR^+^). HLA-DR^+^ immunolabeling in stage I and II granulomas showing: (a&b): strong positive expression observed within the cytoplasm of macrophages (arrows), and multi-nucleated giant cells (green arrow) respectively. (IHC, 50 μm). **(c)**: HLA-DR^+^ macrophages immunolabeling forming a peripheral rim surrounding the necrotic center of the stage III granuloma (arrows). **(d)**: Expression of HLA-DR^+^ macrophages (arrows), in a stage IV granuloma. (IHC, 100 μm). The expression of HLA-DR^+^ macrophages can be seen surrounding the necrotic center in the outer layer of the granuloma. (IHC, 100 μm)

**Fig. 8:**
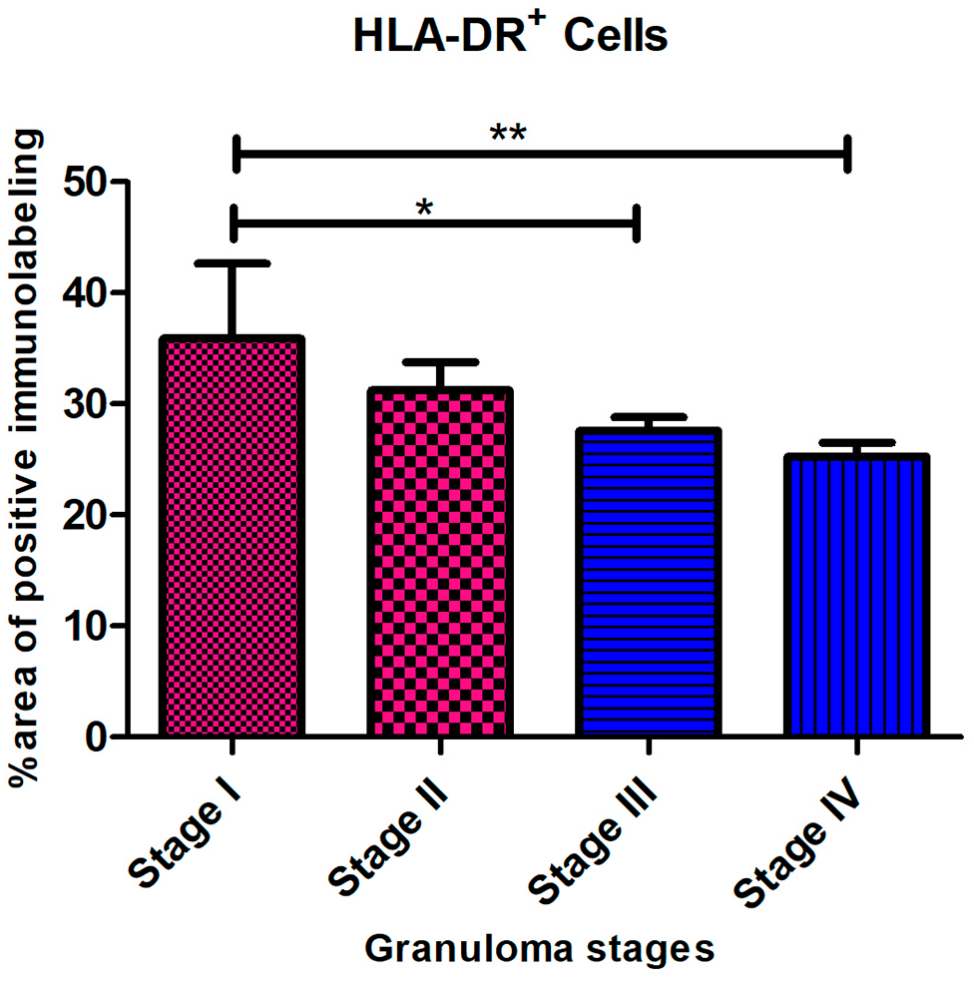
HLA-DR^+^ Cells. The mean percentage of the immunolabeling fraction of HLA-DR^+^ macrophages significantly decreased as the stage of granuloma increased from stage I to stage IV showing similar kinetics as iNOS^+^ cells. The HLA-DR^+^ immunolabeling fraction was higher in the early-stage granulomas compared with the late stages, with a significant difference between stage I and stage II both with respect to stage IV) (** =*P < 0.002)*

Anti-HLA-DR antibody was used to identify activated macrophages. The expression of HLA-DR was observed in all stages of granuloma with differences in the distribution of immunolabeled cells and percentage of positive area in each stage of granuloma. HLA-DR immunolabeling was observed as diffuse intracytoplasmic within abundant epithelioid macrophages and MNGCs distributed throughout stage I and stage II granulomas (Fig. 7a and b). In advanced granuloma stages, central caseous necrosis with mineralization was rimmed predominantly by HLA-DR^+^ macrophages and MNGCs, but also at the peripheral margin of the granuloma (Fig. 7c and d). The number of HLA-DR^+^ cells showed similar kinetics to iNOS^+^ cells and decreased as granuloma development occurred (** =*P < 0.0020)* (Fig. 8).

## Discussion

Bovine TB is a chronic, progressive, infectious, and contagious disease caused by *M. tuberculosis complex* [36]. It is characterized by gross and microscopic lesions (tubercles), however, the antemortem diagnosis of this disease is difficult due to the chronicity of the infection and the modulation of the immune response. A state of anergy and a suppressed cell-mediated immune response may occur in animals in the more advanced stages of the disease, rendering them insensitive to the traditional cell-mediated diagnostic tests (i.e., tuberculin and gamma-interferon) [37]. Post-mortem examination allows the identification of the mycobacteria by different techniques, although there are several factors affecting the sensibility of this diagnosis for example, the relatively high proportion of small lesions which could not be detected in routine inspection. In our study, all cattle examined by visual inspection and histopathology exhibited characteristic gross tubercles and microscopic granulomas, respectively.

Regarding zoonotic diseases, such as bTB, meat inspection and identification of gross lesions at the slaughterhouse are of special importance. Our findings revealed a prevalence of bovine tuberculosis of 2.79% in the imported beef cattle that were under investigation. This finding nearly agrees with previous studies [38, 39], but differs from others with a higher prevalence (11.6–24%) [40–42]. The low prevalence observed in our study may be attributable to the low number of tissues inspected at the slaughterhouse, or depend on other diagnostic techniques, variation in the number of examined animals [42] as well as the lack of detection of small lesions in a routine inspection. In addition, our study was mainly carried out on imported beef cattle considered as a species or subspecies of domestic cattle, zebu, which is relatively resistant to bTB [43]. However, a more recent study showed no difference between Zebu and Holstein-Friesian cattle [44]. Indeed, some of the previous studies with a higher prevalence rate were conducted on animals belonging to other breeds than zebu [40, 41]. Therefore, our findings support that the slaughterhouse meat inspection procedures represent a critical point for the identification of TBL gross lesions at the slaughterhouse. It is advisable to enhance meat inspection procedures such as multiple slicing of organs and lymph nodes in young calves (< 8 months [45, 46].

In this study, most of the tuberculosis cases were diagnosed as localized tuberculosis (51, 85%), especially in a pulmonary form where the lesion was frequently seen in the lungs and lymph nodes of the thoracic cavity (complete primary complex). This finding revealed that these imported cattle harbored already the infection from the country of origin. It has been described that chronically infected animals showing severe gross pathology may be unresponsive to the tuberculin test yielding a false negative result [9, 47]. Therefore, it is very important to use additional diagnostic tools to confirm the bTB status of imported beef cattle. The polymerase chain reaction (PCR) is a powerful technique that is considered one of the most widely used techniques in the diagnosis of *Mycobacterium tuberculosis* complex infections through the amplification of different targets, such as IS*6110* [48]. In addition, a histopathological examination is a diagnostic tool with an acceptable sensitivity (87.5%) and specificity (84.1%) which can be used both to confirm the diagnosis obtained by skin intra-dermal tuberculin test and to identify true positive animals which disclose negative results to the other techniques [13]. However, these techniques are currently applied postmortem which impedes having a diagnosis before the movement of animals. In spite of all samples having characteristics of tuberculosis like lesions grossly and presented the characteristic tuberculous granuloma, including granulomas from different stages in every sample microscopically, mycobacterial isolation was not possible in all of them due to some limitations such as a low number of AFB in samples where most of ZN^+^ samples presented a paucibacillary form (83.33%; 35/42) or even complications related to the high level of natural contamination. In the study, out of 60 cultures tested, 35 (58%; 35/60) were positive. Our findings are higher than those reported by Proano-Perez et al., (2011) (36.4%) [49] and nearly similar to those reported by Mecherouk et al., (2023) [50].

To further comprehend the function and distribution of macrophages in calves naturally infected with tuberculosis, this study used macroscopic lesions, histological assessment of granulomas, and immunohistochemistry analyses. In the present study, we selected samples from lymph nodes to study the immune response of macrophages at different stages of granuloma. The criteria of these selection were based on presence of the four granuloma stages in lymph node samples. In addition, in some cases, the lesions were detected in the regional lymph nodes related to the lungs only, therefore these cases may be incomplete primary complexes [51] where the lesion was detected in the lymph nodes only.

Our findings show that the frequency of immunolabeled cells against these markers progressively reduced from stage I to stage IV granuloma. However, other authors have mentioned a lack of changes or even an increase in the number of CD68^+^ cells with lesion progress in cattle [10, 12, 26]. CD68 expression was observed within the cytoplasm of macrophages, epithelioid macrophages, and Langhans MNGCs at all stages of granuloma. The number of CD68^+^ cells dropped as the granuloma developed. CD68 belongs to the lysosomal/endosomal-associated membrane glycoprotein (LAMP) family, which is involved in antigen processing and presentation during phagocytosis [52]. This could be associated with the role of phagocytic activity of macrophages and MNGCs in the early immune response for protection and elimination of the mycobacteria and might indicate a decrease in the phagocytic activity in the later stages of the disease.

iNOS expression followed a similar trend as CD68^+^ cells, with iNOS^+^ macrophages seen as dispersed throughout stages I and II granulomas and in a distinct ring encircling the necrotic core in stages III and IV granulomas. Similar kinetics for iNOS^+^ and CD68^+^ macrophages have been reported in previous studies [11, 26]. It is hypothesized that increased nitric oxide (NO) production by iNOS^+^ macrophages is crucial for the management and eradication of mycobacteria during infection [11, 26]. NO inactivates mycobacteria by producing highly reactive nitrogen intermediates [26]. Therefore, NO generation during tuberculosis may increase due to the increased iNOS^+^ macrophage population. The NO decreased the number of bacteria and cleared the infection [24, 26], more importantly in early-stage granulomas. Furthermore, the similar kinetics observed concerning CD68 expression support a decrease of macrophage (M 1) polarization from the early stages to the later stages of the disease.

According to Heppner et al. (2015) [21], HLA-DR is a macrophage marker crucial for controlling immunological responses and antigen presentation, particularly for T cell-mediated reactions. Our findings showed that early-stage granulomas (I, II) had higher expression of HLA-DR^+^ and iNOS^+^ than late-stage granulomas (III, IV). Increased iNOS expression has been associated with higher levels of IFN-γ in granulomas of calves who received the BCG, favoring those with a better host immune response against mycobacteria [26]. IFN-γ is a Th1 cytokine known to stimulate or activate macrophages, developing mycobactericidal mechanisms [53]. The parallel increase in the expression of CD68, iNOS, and HLA-DR in early-stage granulomas and their progressive decrease in late-stage granulomas indicate a shift in the immune response along the infection, from a more effective proinflammatory immune response to a tolerogenic response which allows the persistence of the disease. Furthermore, the similar kinetics observed with respect to iNOS expression support a decrease of macrophage (M 1) polarization from the early stages to the later stages of the disease.

## Conclusion

This study elucidated the role and expression of CD68^+^, iNOS^+,^ and HLA-DR^+^ macrophages in different stages of the granuloma in naturally infected cattle. The increased expression of CD68^+^, iNOS^+,^ and HLA-DR^+^ macrophages in early-stage granulomas (I, II) compared with late-stage granulomas (III, IV) suggests that macrophages are initially polarized to pro-inflammatory macrophages, playing a protective role in the immune response during the early stages of the disease in naturally infected beef cattle.

## Data Availability

The data supporting this study’s findings are available on request from the corresponding author.

## Abbreviations

ABC: Avidin-biotin-peroxidase complex
ANOVA: Analysis of variance
AFB: Acid-fast bacilli
bTB: Bovine tuberculosis
CMI: Cell-mediated immune
H&E: Hematoxylin-eosin
HLA-DR: Human leucocytic antigen-DR
HPF: High-power field magnification
iNOS: Inducible nitric oxide synthase
MHC-II: Class II MHC antigens
MNGCs: Multinucleated giant cells
MTC: Mycobacterium tuberculosis complex
NO: Nitric oxide
PBS: Phosphate buffer saline
PCR: Polymerase chain reaction
SD: Standard deviation
TBL: Tuberculosis-like lesions
Th1: T helper type 1
WHO: World Health Organization
ZN: Ziehl-Neelsen
